# Impact of tight blood glucose control on atrial fibrillation in critically ill patients receiving early parenteral nutrition: an individual patient data meta-analysis of two large randomized controlled trials

**DOI:** 10.1186/s13019-026-04066-0

**Published:** 2026-04-15

**Authors:** Erwin De Troy, Jan Gunst, Pieter J. Wouters, Greet Van den Berghe, Dieter Dauwe

**Affiliations:** Clinical Division and Laboratory of Intensive Care Medicine, Department of Cellular and Molecular Medicine, KU Leuven, Leuven, B-3000 Belgium

**Keywords:** Critical illness, Atrial fibrillation, Tight glucose control, Hyperglycemia, Insulin

## Abstract

**Background:**

Atrial fibrillation often occurs during critical illness. Tight glucose control with insulin (TGC) is known to reduce inflammation and oxidative stress and may alter atrial metabolism, which, together, could affect atrial fibrillation pathogenesis. Our group has previously shown that TGC reduced morbidity and mortality in a mixed medical/surgical critically ill patient population receiving early parenteral nutrition as part of the contemporary standard of care. We here hypothesized that TGC reduces atrial fibrillation in the intensive care unit (ICU).

**Methods:**

In this individual patient data meta-analysis of 2 randomized controlled trials (performed 2000–2001 and 2002–2005), we investigated the impact of TGC with insulin (targeting blood glucose 80–110 mg/dL) in comparison with tolerating hyperglycemia to 215 mg/dL (liberal glucose control [LGC]) on atrial fibrillation in mixed surgical (*n* = 1548) and medical (*n* = 1200) ICU patients admitted to a quaternary-care university hospital. Atrial fibrillation was further classified as new-onset or recurrent/persistent pre-existing atrial fibrillation. The primary endpoint was the impact of TGC on atrial fibrillation in ICU, determined via multivariable logistic regression analysis after adjusting for relevant baseline patient characteristics. Prespecified subgroup analyses were performed for patients with a history of diabetes mellitus, pre-existing atrial fibrillation, overall surgical admission, admission after cardiac surgery and for patients with an ICU-stay longer than 5 days, after assessing treatment heterogeneity via determination of interaction p-values.

**Results:**

Atrial fibrillation in ICU occurred in 845/2639 patients (32.0%), 65.9% of which was new-onset atrial fibrillation. TGC had no impact on atrial fibrillation in ICU (adjusted OR 0.92 [0.77–1.11])(*P* = 0.40). TGC also did not affect new-onset atrial fibrillation (adjusted OR 0.92 [0.75–1.12])(*P* = 0.39). There was no treatment heterogeneity present for the pre-defined subgroups except for the subgroup of patients with history of diabetes mellitus (*n* = 390), in which atrial fibrillation was documented for 66/199 patients in the TGC-group (33.2%) and 80/191 patients in the LGC-group (41.9%), interaction *P* = 0.045). Atrial fibrillation in ICU was strongly associated with worse outcome.

**Conclusions:**

TGC in the context of early use of parenteral nutrition did not reduce atrial fibrillation during ICU-stay in this large mixed medical-surgical ICU cohort. A possible exception was noted for patients with a history of diabetes mellitus.

**Supplementary Information:**

The online version contains supplementary material available at 10.1186/s13019-026-04066-0.

## Background

Atrial fibrillation is common in intensive care unit (ICU) patients and the most common cardiac arrhythmia in this population which can occur as new-onset atrial fibrillation or as recurrent/persistent pre-existing atrial fibrillation. In ICU patients, the prevalence ranges from 10% to 60%, with the highest prevalence reported for patients admitted after cardiac surgery [1–7]. The occurrence of atrial fibrillation in the ICU has been associated with prolonged ICU and hospital length of stay as well as increased short-term mortality, and patients with atrial fibrillation in ICU have an increased long-term risk of atrial fibrillation recurrence, heart failure, stroke, and mortality [2, 8–14].

The high prevalence of atrial fibrillation in ICU patients could be triggered by adrenergic activation, electrolyte and metabolic disturbances, volume overload, systemic inflammation, and hypoxia/ischaemia, among others [15, 16]. In cardiac surgery patients, myocardial and pericardial inflammation, as well as oxidative stress, may further increase susceptibility for atrial fibrillation [15, 17]. As critical illness is accompanied by a metabolic stress response characterized by insulin resistance and stress hyperglycemia as well as hypertriglyceridemia, altered substrate metabolism may also play a role in the pathogenesis of ICU-related atrial fibrillation [18, 19]. These potential triggers for atrial fibrillation may or may not initiate atrial fibrillation during ICU stay depending on the extent of the underlying electrical, structural or metabolic remodeling within the atrial myocardium [20]. In this context, subgroups of ICU patients could be particularly vulnerable for the development of atrial fibrillation. As suggested by available literature, these include patients with pre-existing atrial fibrillation, patients admitted after surgery, in particular those admitted after cardiac surgery and patients with a history of diabetes mellitus [4, 7, 21]. Also, long-stay ICU patients, who are exposed for a longer period of time to metabolic abnormalities such as hyperglycemia or dyslipidemia, may be a vulnerable subgroup [22–25].

Consequently, the simultaneous presence of various local and systemic triggers on top of a vulnerable substrate likely explains the higher prevalence of atrial fibrillation after cardiosurgical procedures as compared to medical admissions. Indeed, the surgical ‘trauma’ is associated with intramyocardial and intrapericardial inflammation, and the underlying disease requiring cardiac surgery is often associated with atrial remodeling, altogether lowering the threshold for atrial fibrillation [16, 17].

Our group has previously shown that tight glucose control with intensive insulin therapy (TGC) significantly improved morbidity and mortality of ICU patients receiving early parenteral nutrition, as compared with tolerating hyperglycemia up to the renal threshold [22, 23]. Mechanistic studies attributed these benefits to prevention of cellular glucose overload and associated mitochondrial damage in vital organs including the myocardium and to partial restoration of the dyslipidemia [25–28]. Moreover, the intervention reduced inflammation and oxidative stress, which could affect atrial fibrillation pathogenesis [29–31]. Furthermore, the impact of TGC on vital organ dysfunction was more pronounced in patients receiving early parenteral nutrition than in patients who did not receive early parenteral nutrition, possibly explained by more severe hyperglycemia causing more cellular damage in a context of macronutrient-driven suppression of cellular repair systems such as autophagy [32]. However, the impact of TGC on atrial fibrillation in ICU has hitherto not been thoroughly studied. A meta-analysis suggested that TGC reduced atrial fibrillation in ICU, although data were only available from a few small studies performed in cardiac surgery patients [33].

In the current individual patient data meta-analysis of 2 large randomized controlled trials (RCTs), we investigated the effect of TGC on atrial fibrillation in ICU for a mixed medical-surgical ICU population receiving early parenteral nutrition as part of the contemporary standard of care, adjusted for baseline characteristics. We hypothesized that for these critically ill patients, TGC with insulin, while providing exogenous metabolic substrates via early supplemental parenteral nutrition, reduces atrial fibrillation in ICU. Prespecified subgroup analyses were also performed for those patients with known vulnerability for atrial fibrillation in ICU. Furthermore, the associations between atrial fibrillation in ICU and outcomes were determined.

## Methods

### Study population and design

This is an individual patient data meta-analysis of 2 single-centre RCTs performed from 2000 to 2001 in the adult surgical ICU and from 2002 to 2005 in the adult medical ICU of the University Hospitals Leuven, Leuven, Belgium. The study protocol and primary results have been published previously [22, 23]. In brief, patients admitted to the ICU were randomly assigned to tight glucose control with insulin (TGC), targeting blood glucose concentrations between 80 and 110 mg/dL (4.4–6.1 mmol/L) with the insulin infusion titrated making use of a syringe pump and connected to a central venous catheter, or to liberal glucose control (LGC) with insulin only administered when blood glucose concentrations exceeded 215 mg/dL (11.9 mmol/L) and insulin stopped when blood glucose concentrations were below 180 mg/dL (10.0 mmol/L). All patients received early parenteral nutrition to supplement insufficient or failing enteral nutrition as part of the contemporary standard of care in these ICUs.

The presence of atrial fibrillation while patients were in the ICU (further referred to as ‘atrial fibrillation in ICU’) was determined based on predefined objective criteria, derived from the medical records and the use of specific medication to treat atrial fibrillation during the ICU stay. A patient was considered to have had an episode of atrial fibrillation during ICU stay if atrial fibrillation was mentioned in the medical record, and/or if specific medications to treat atrial fibrillation (amiodarone, digoxin, mexiletine, propafenon, verapamil, sotalol, diltiazem, flecainide) were administered in the absence of documented other indications (including cardiopulmonary resuscitation, atrial flutter and other non-atrial fibrillation supraventricular tachycardias, ventricular fibrillation, ventricular tachycardia, continuation of pre-admission drugs and prophylaxis). The presence of pre-existing atrial fibrillation was derived from the medical history and all available pre-admission 12-lead electrocardiograms. Based on this information, atrial fibrillation during ICU stay was further classified as new-onset atrial fibrillation or recurrent/persistent pre-existing atrial fibrillation.

The primary endpoint was the impact of TGC versus LGC on atrial fibrillation in ICU. Prespecified subgroup analyses were performed for patients with pre-existing atrial fibrillation, pre-existing diabetes, surgical versus medical ICU patients and patients admitted after cardiac surgery. Another a priori defined subgroup analysis was performed for patients in ICU for more than 5 days as this subgroup was previously shown to benefit most from TGC [22]. The occurrence of new-onset atrial fibrillation during ICU stay was studied as a secondary endpoint. Patients with no information on preexisting atrial fibrillation were excluded for analysis of this secondary endpoint.

Additionally, we investigated the association between atrial fibrillation in ICU and patient-centered outcome parameters, including ICU mortality, hospital mortality, ICU length of stay, duration of ventilatory support, renal impairment in ICU (defined by serum creatinine peak value and the need for renal replacement therapy), inflammation as indicated by the C-reactive protein (CRP) peak value in ICU, bacteremia in ICU and number of days on inotropic or vasopressor support.

The RCTs were performed in accordance with the 1964 Declaration of Helsinki and later amendments. The protocols and consent forms were approved by the institutional ethical review board. Written informed consent was obtained from the closest family member.

### Statistical analyses

Individual patient data from the two RCTs were pooled into one database. Continuous and categorical variables are presented as median (interquartile range) and number (percent), respectively.

Univariable analyses of differences between patient groups were performed using Mann-Whitney U test for continuous variables and Chi-square or Fisher’s Exact test for categorical variables, as appropriate.

The impact of TGC versus LGC on atrial fibrillation in ICU was determined via multivariable logistic regression analysis adjusting for baseline patient characteristics including age, sex, body-mass index (BMI), history of diabetes mellitus (DM), history of hypertension, history of atrial fibrillation, history of heart failure, the severity of illness scores Therapeutic Intervention Scoring System-28 (TISS-28) score and Acute Physiology And Chronic Health Evaluation II (APACHE II) score, and surgical versus medical reason for admission.

To study potential heterogeneity of treatment effects in the predefined subgroups, an interaction term was added to the multivariable model. Treatment heterogeneity between the different subgroups was visualized by forest plot. Odds ratios (OR) are reported with 95% confidence intervals. For interaction P-values, the threshold for statistical significance was set at 0.1, in order not to miss a potential interaction that would be clinically relevant. For other comparisons, two-tailed P-values below 0.05 were considered statistically significant. No adjustments for multiplicity or imputations for missing data were performed. All analyses were performed with the use of JMP software, version pro 17.0.0 (SAS Institute, NC, USA).

## Results

### Study population

**Fig. 1:**
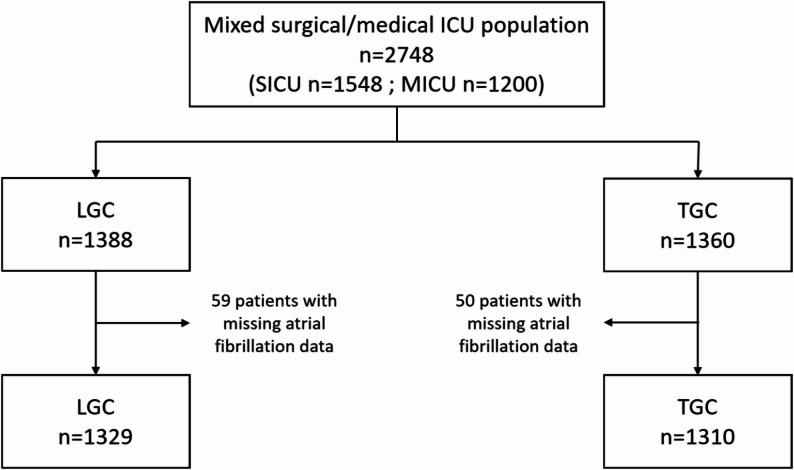
CONSORT diagram. CONSORT diagram summarizing patient selection and inclusion process. ICU, intensive care unit; SICU, surgical intensive care unit; MICU, medical intensive care unit; LGC, liberal glucose control; TGC, tight glucose control

Of 2748 patients who had been included in the 2 RCTs, data on atrial fibrillation in ICU were missing for 109 patients, equally distributed across the TGC and the LGC group. Hence, 2639 patients were included for this study, 1310 randomly allocated to the TGC group and 1329 to the LGC group (Fig. 1). Baseline patient characteristics were largely comparable for the two randomization groups, except for pre-existing heart failure and body-mass index (Table 1).

In the current study population, the impact of TGC versus LGC on mortality and morbidity other than atrial fibrillation in ICU, was similar to what has been reported earlier for the total patient population (Supplementary Material 1: Supplementary Table S1) [24]. Baseline patient characteristics according to surgical versus medical ICU admission can be found in the supplementary file (Supplementary Material 1: Supplementary Table S2).

**Table 1:** Demographic and clinical characteristics of the patients at baseline

Characteristic	Total population *n* = 2639	LGC *n* = 1329 (50.4%)	TGC *n* = 1310 (49.6%)	*P*-value
Age (years) – median (IQR)	66 (55–74)	65 (55–74)	66 (55–74)	0.36
Male gender – n (%)	1767 (67.0%)	903 (68.0%)	864 (66.0%)	0.28
Diabetes mellitus – n (%)	390 (14.8%)	191 (14.4%)	199 (15.2%)	0.58
Hypertension * – n (%)	695 (28.1%)	347 (27.7%)	348 (28.4%)	0.75
Heart failure * – n (%)	282 (11.4%)	162 (12.9%)	120 (9.8%)	0.01
Pre-existing atrial fibrillation * – n (%)	417 (16.7%)	215 (17.0%)	202 (16.3%)	0.67
BMI (kg/m^2^) * – median (IQR)	25 (23–28)	24.7 (22–28)	24.9 (23–28)	0.03
APACHE II * – median (IQR)	12 (8–18)	12 (8–18)	12 (8–18)	0.29
TISS-28 first 24 h * – median (IQR)	36 (29–44)	36 (29–44)	37 (29–44)	0.52
Blood glucose on admission (mg/dL) – median (IQR)	137 (110–173)	138 (110–177)	135 (109–169)	0.07
SICU – n (%)	1517 (57.5%)	764 (57.5%)	753 (57.5%)	1.00
Admission after cardiac surgery – n (%)	956 (36.2%)	487 (36.6%)	469 (35.8%)	0.66

Data are presented as frequencies and percentages or medians with interquartile ranges

LGC, liberal glucose control; TGC, tight glucose control; BMI, body mass index; APACHE II, Acute Physiology and Chronic Health Evaluation II; TISS-28, Therapeutic Intervention Scoring System-28; SICU, surgical intensive care unit

* Missing data on baseline characteristics: hypertension: *n* = 161; heart failure: *n* = 158; pre-existing AF: *n* = 139; BMI: *n* = 40; APACHE II: *n* = 8; TISS-28: *n* = 27

### Impact of TGC on atrial fibrillation

Atrial fibrillation in ICU occurred in 845 of 2639 patients (32.0%). New-onset atrial fibrillation occurred in 534 of 2500 patients (21.4%) (data regarding pre-existing atrial fibrillation were missing in 139 patients). Baseline patient characteristics significantly associated with atrial fibrillation in ICU were higher age, history of diabetes mellitus, history of hypertension, history of heart failure, history of atrial fibrillation, a higher body-mass index, higher severity of critical illness scores and admission after cardiac surgery (Supplementary Material 1: Supplementary Table S3).

Atrial fibrillation in ICU was present in 31.3% (410/1310) of patients in the TGC group and 32.7% (435/1329) of patients in the LGC group. After adjustment for baseline patient characteristics, TGC had no effect on atrial fibrillation in ICU: adjusted OR 0.92 [95% confidence intervals 0.77–1.11], *P* = 0.40 (Table 2; Supplementary Material 1: Supplementary Table S4). TGC also did not affect new-onset atrial fibrillation, which occurred in 21.0% (259/1236) of patients in the TGC group and 21.8% (275/1264) of patients in the LGC group: adjusted OR 0.92 [0.75–1.12], *P* = 0.39.

**Table 2:** Impact of TGC on atrial fibrillation during ICU stay

Outcome parameter	LGC *n* = 1329 (50.4%)	TGC *n* = 1310 (49.6%)	Adjusted OR (95% CI)	Adjusted *P*-value
PRIMARY OUTCOME				
Overall atrial fibrillation in ICU	435/1329 (32.7%)	410/1310 (31.3%)	0.92 (0.77–1.11)	0.40
SECONDARY OUTCOME				
New-onset atrial fibrillation in ICU *	275/1264 (21.8%)	259/1236 (21.0%)	0.92 (0.75–1.12)	0.39

ICU, intensive care unit; LGC, liberal glucose control; TGC, tight glucose control

* Missing data on pre-existing atrial fibrillation: *n* = 139

**Fig. 2:**
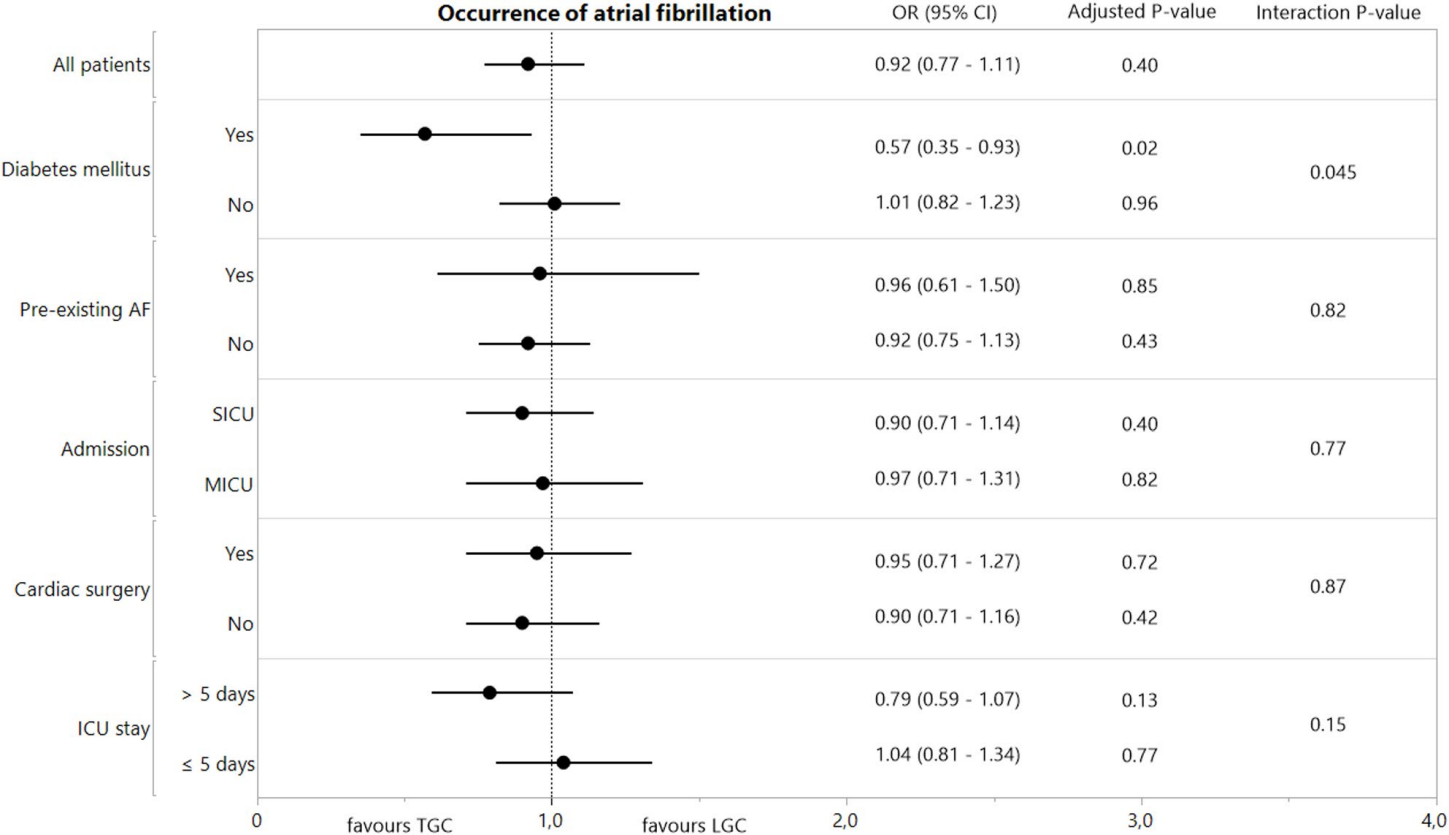
Impact of TGC on atrial fibrillation during ICU stay in prespecified subgroups. The panel shows a forest plot of the impact of tight glucose control versus liberal glucose control in subgroups with respect to the development of atrial fibrillation during ICU stay. Odds ratios and 95% confidence intervals, P-values and interaction P-values are all adjusted analyses. SICU: surgical intensive care unit; MICU: medical intensive care unit; ICU: intensive care unit

Interaction analyses did not reveal treatment heterogeneity for the impact of TGC on atrial fibrillation in ICU for the predefined subgroups of patients with pre-existing atrial fibrillation, surgical vs. medical ICU admission, patients admitted after cardiac surgery and for patients with an ICU length of stay of more than 5 days (Fig. 2). In contrast, potential heterogeneity of treatment effect was observed for patients with a history of diabetes mellitus versus patients without such a history (interaction *P* = 0.045). Among patients with a history of diabetes mellitus, atrial fibrillation occurred in 66/199 TGC group patients (33.2%) and in 80/191 LGC group patients (41.9%) (*P* = 0.02). Such a TGC-effect in the subgroup of patients with a history of diabetes mellitus was not present for new-onset atrial fibrillation in ICU (Supplementary Material 1: Supplementary Table S5 and Supplementary Figure S1).

### Association of atrial fibrillation in ICU with morbidity and mortality

The presence of atrial fibrillation in ICU was associated with increased morbidity and mortality (Supplementary Material 1: Supplementary Table S6). Patients with atrial fibrillation had a significantly longer duration of mechanical ventilation (5 [2–12] vs. 2 [1–6] days, *P* < 0.01), a higher peak creatinine concentration (1.47 [1.07–2.92] vs. 1.13 [0.90–1.97] mg/dL, *P* < 0.01), a higher need for renal replacement therapy (22.6% vs. 7.6%, *P* < 0.01), more inflammation (CRP peak value 219 [148–287] mg/L vs. 168 [88–248] mg/L, *P* < 0.01), a higher incidence of bloodstream infections (10.4% vs. 5.0%, *P* < 0.01), more days on inotropic support (2 [0–4] vs. 0 [(0–2] days, *P* < 0.01) and vasopressor support (2 [0–5] vs. 0 [0–2] days, *P* < 0.01). Patients with atrial fibrillation during ICU stay also had a significantly longer ICU length of stay (6 [3–15] vs. 3 [2–7] days, *P* < 0.01), and a higher ICU mortality (23.6% vs. 10.8%, *P* < 0.01) and hospital mortality (32.7% vs. 17.0%, *P* < 0.01).

## Discussion

In this individual patient data meta-analysis of two large RCTs on TGC in the context of early administration of parenteral nutrition, atrial fibrillation occurred in approximately one third of mixed surgical/medical critically ill patients and was associated with worse clinical outcomes. Lowering blood glucose levels to the healthy fasting range with insulin, as compared with accepting severe hyperglycemia, did not affect the occurrence of atrial fibrillation during ICU stay. A possible exception was noted for the subgroup of patients with diabetes, in whom TGC appeared to reduce the risk of atrial fibrillation. Various other pre-defined subgroup analyses in this large and heterogeneous study population did not document heterogeneity of treatment effect.

Up to date, no large RCT has studied the effect of TGC on the occurrence of atrial fibrillation during ICU stay. A limited number of smaller RCTs have reported the effect of TGC on postoperative atrial fibrillation, which showed conflicting results [34–37]. However, when pooled together in two meta-analyses, a signal towards a reduction of postoperative atrial fibrillation by TGC provided during the perioperative period, was suggested [33, 38]. Of note, apart from small sample size (majority ≤ 200 patients), included studies had important heterogeneity in design, both in blood glucose target as well as timing and duration of tight glucose control. Moreover, all studies were performed in a surgical ICU population. In contrast, we performed an individual patient data meta-analysis in a large dataset (2639 patients) of two RCTs in a mixed surgical/medical ICU cohort, using a uniform protocol of TGC with insulin during ICU stay.

We did not detect an impact of TGC on the occurrence of atrial fibrillation during ICU stay in the overall population. In contrast to previous RCTs, which studied the effect of TGC on postoperative atrial fibrillation, mainly after cardiac surgery, we also included a large cohort of 1122 patients after medical ICU admission. We did not detect an impact of TGC on atrial fibrillation in a large subgroup of patients admitted to the ICU after cardiac surgery (956 patients) or after surgical admission in general (1517 patients).

Our study was performed in the context of early parenteral nutrition, as part of the contemporary standard of care when these RCTs were conducted [22, 23]. Subsequently, it was shown that withholding parenteral nutrition until one week after admission, which lowers the risk and severity of hyperglycemia, improved recovery from critical illness [39]. The impact of the early feeding strategy on the occurrence of atrial fibrillation during ICU stay still needs to be investigated. Whether our results regarding the impact of TGC on atrial fibrillation can be extrapolated to the context of withholding parenteral nutrition during the first week of ICU stay as the current evidence-based standard of care, still needs to be studied. In the recent TGC-Fast trial, TGC with insulin did not alter the length of time that ICU care was needed or mortality in the context of withholding parenteral nutrition during the first week of ICU [32]. However, the intervention was associated with a significant reduction in severe acute kidney injury and cholestatic liver dysfunction, suggesting a potential morbidity benefit. Importantly, in TGC-Fast, TGC was advised by a validated protocol that avoided hypoglycemia. The NICE-SUGAR trial showed increased mortality by TGC in patients not receiving early parenteral nutrition, with a protocol that led to a 14-fold increased risk of severe hyperglycemia [40]. Hence, the ideal blood glucose target likely depends on the context [41]. Contemporary guidelines recommend to prevent at least severe hyperglycemia (< 180 mg/dL), and to consider tighter control only if it can be achieved without inducing hypoglycemia [42].

Accepting an early caloric deficit activates cellular repair mechanisms, such as autophagy [43]. Moreover, the severity of hyperglycemia with consequent glucose toxicity is less pronounced, while systemic inflammation has been shown to be intensified [39, 44]. The combined impact of all of these factors known to be involved in atrial fibrillation pathogenesis, on the initiation and perpetuation of atrial fibrillation during ICU stay is currently unknown.

As previously reported, the incidence of hypoglycemia as well as the extent of glycemic variability (assessed as the mean daily difference between minimum and maximum blood glucose), was markedly higher in the TGC group compared to the LGC group [22, 23, 45]. Hypoglycemia has been shown to be an independent risk factor for atrial fibrillation in non-ICU patients with type 2 diabetes [46]. Glycemic variability is linked to increased oxidative stress, inflammation and apoptosis, all of which are involved in the pathogenesis of atrial fibrillation [47]. High glycemic variability was indeed associated with a higher risk of atrial fibrillation in a meta-analysis including patients with acute coronary syndromes and patients undergoing coronary artery bypass grafting [48]. Whether the higher incidence of hypoglycemia and the increased glycemic variability in the TGC group could have outbalanced a potential beneficial effect of TGC on the occurrence of atrial fibrillation during ICU stay is currently unknown. The recent TGC-Fast trial reported very few hypoglycemic events in both treatment groups (≤ 1.0%), with use of a computer-based insulin-infusion algorithm (LOGIC-Insulin) to guide insulin infusion [32]. Whether this could impact the effect of TGC on atrial fibrillation during ICU stay remains to be studied.

This individual patient data meta-analysis has some strengths and weaknesses. A major strength of the present study is that up to date, this is the largest randomized dataset investigating the effect of a uniform TGC algorithm on the occurrence of atrial fibrillation in a mixed ICU population. As this study was performed in a large and heterogeneous, critically ill patient population, it also allowed assessment of the heterogeneity in treatment effect in large subgroups. Potential treatment heterogeneity was limited to the subgroup of patients with a history of diabetes mellitus (390 patients), in whom TGC appeared to reduce the prevalence of atrial fibrillation during ICU stay. Patients with diabetes mellitus are particularly vulnerable to the development of atrial fibrillation during critical illness. Diabetic (atrial) cardiomyopathy provides a marked substrate for the initiation and perpetuation of atrial fibrillation. Factors involved in the development of this proarrhythmogenic substrate are multiple and include increased oxidative stress, low grade inflammation, as well as insulin resistance with resulting altered cardiomyocyte substrate metabolism, characterized by increased reliance on fatty acid over glucose metabolism [49–51]. TGC with insulin has been shown to have a favourable impact on all of these contributing pathogenetic mechanisms [52]. The interference of the intervention with multiple of these highly pronounced pathways in atrial fibrillation pathogenesis may explain why TGC was associated with reduced atrial fibrillation during ICU stay exclusively in patients with a history of diabetes mellitus. Moreover, early administration of parenteral nutrition may have exacerbated stress-induced hyperglycemia, particularly in patients with pre-existing diabetes. This effect could have contributed to the observed benefit of TGC on atrial fibrillation within this subgroup. Hence, these findings may provide additional rationale for withholding early parenteral nutrition during critical illness. However, our trial was not powered to study TGC-mediated effects in specific subgroups, hence these results remain exploratory.

A weakness of the present study regards the modality of data extraction for detecting atrial fibrillation. The presence of atrial fibrillation in ICU, as well as its classification as new-onset or recurrent/persisting pre-existing atrial fibrillation, was derived from patient records, medications and available electrocardiograms, which may have provoked both over- and underdiagnosis of episodes. Access to high temporal resolution data from telemetry was not available, which is the ‘gold standard’ for atrial fibrillation detection and analysis regarding timing, as well as number and duration of atrial fibrillation episodes. Nevertheless, our reported prevalences of atrial fibrillation during ICU stay in the overall population as well as in specific subgroups, are in line with what has been reported in current literature [4, 21, 53]. Moreover, our reported associations of atrial fibrillation during ICU stay with baseline risk factors and short-term outcomes are in agreement to what is expected and previously reported [4, 53]. There was a slight baseline imbalance in baseline characteristics. Although multivariable models were adjusted for baseline characteristics, we cannot exclude residual confounding.

This is an individual patient data meta-analysis of two large RCTs performed between 2000 and 2005. Since then, overall clinical practice in terms of surgical techniques and medical ICU management has evolved. This includes an overall shift towards more restrictive use of parenteral nutrition in the early phase of critical illness, with, consequently, less severe hyperglycemia [54]. Whether these results regarding the impact of TGC on atrial fibrillation still hold true in the current ICU context, still needs to be studied. However, also in current ICU practice, patients often present with severe hyperglycemia, in particular in the presence of other, iatrogenic causes such as corticosteroids.

We only studied atrial fibrillation during ICU stay. Data on atrial fibrillation after ICU discharge to the conventional hospital ward was not available. Since approximately one third of the patients had an ICU length of stay shorter than three days, and the peak incidence of atrial fibrillation is typically during the first 2–3 days after surgery or ICU admission, a possible effect of TGC on atrial fibrillation in this subpopulation may have been be missed [13, 55]. Moreover, it is known that patients experiencing atrial fibrillation during ICU stay have an increased long-term risk of atrial fibrillation recurrence [21]. As long-term outcome data were not available in our study, we were not able to assess whether TGC with insulin during ICU stay had a lasting impact on atrial fibrillation initiation, recurrence or perpetuation after hospital discharge.

## Conclusion

In the context of early use of parenteral nutrition, lowering blood glucose levels into the healthy fasting range with insulin in a mixed surgical/medical cohort of critically ill patients did not affect the risk of atrial fibrillation during ICU stay. A possible exception was noted for the prespecified subgroup of patients with a history of diabetes mellitus, in whom TGC appeared to reduce the risk of atrial fibrillation in the ICU.

## Supplementary Information

Below is the link to the electronic supplementary material.

Supplementary Material 1

## Data Availability

Deidentified individual participant data used and/or analysed during the current study is available from the corresponding author on reasonable request.

## Abbreviations

TGC: Tight glucose control
ICU: Intensive care unit
LGC: Liberal glucose control
RCT: Randomized controlled trial
OR: Odds ratio
SICU: Surgical intensive care unit
MICU: Medical intensive care unit
CRP: C-reactive protein
