# Supplementary material for: Impact of tight blood glucose control on atrial fibrillation in critically ill patients receiving early parenteral nutrition: an individual patient data meta-analysis of two large randomized controlled trials
Source: J Cardiothorac Surg. 2026 Apr 15;21:276. doi: 10.1186/s13019-026-04066-0 (PMC13214420; doi:10.1186/s13019-026-04066-0)
Supplement: Supplementary file 1 — Supplementary Material 1 [file 13019_2026_4066_MOESM1_ESM.docx]

**Supplementary Information**

**Impact of tight blood glucose control on atrial fibrillation in critically ill patients receiving early parenteral nutrition: an individual patient data meta-analysis of two large randomized controlled trials**

Erwin De Troy, Jan Gunst, Pieter J. Wouters, Greet Van den Berghe, Dieter Dauwe

**Table of contents**

- **Supplementary Table S1** Impact of TGC versus LGC on mortality and short-term outcomes other than atrial fibrillation
- **Supplementary Table S2** Demographic and clinical baseline characteristics according to surgical versus medical ICU admission
- **Supplementary Table S3** Demographic and clinical baseline characteristics according to atrial fibrillation diagnosis during ICU stay
- **Supplementary Table S4** Impact of TGC on atrial fibrillation during ICU stay – multivariable analysis
- **Supplementary Table S5** Impact of TGC on new-onset atrial fibrillation during ICU stay – multivariable analysis
- **Supplementary Figure S1** Impact of TGC on new-onset atrial fibrillation during ICU stay in prespecified subgroups
- **Supplementary Table S6** Association of atrial fibrillation during ICU stay with ICU morbidity and short-term outcomes

**Supplementary Table S1** Impact of TGC versus LGC on mortality and short-term outcomes other than atrial fibrillation

| **Characteristic** | **Total population**  **n=2639** | **LGC**  **n=1329 (50.4%)** | **TGC**  **n=1310 (49.6%)** | **P-value** |
| --- | --- | --- | --- | --- |
| Duration of ventilatory support during ICU stay - median (IQR) | 2 (1-9) | 3 (1-10) | 2 (1-8) | 0.13 |
| Renal impairment during ICU stay |  |  |  |  |
| Peak creatinine value (mg/dl) - median (IQR) | 1.20 (0.94-1.97) | 1.22 (0.95-2.03) | 1.18 (0.93-1.91) | 0.06 |
| Renal replacement therapy – n (%) | 327 (12.4%) | 175 (13.2%) | 152 (11.6%) | 0.24 |
| CRP peak value* (mg/dl) - median (IQR) | 186 (106-266) | 188 (106-271) | 183 (106-258) | 0.56 |
| ICU bacteremia – n (%) | 179 (6.8%) | 102 (7.7%) | 77 (5.9%) | 0.07 |
| Number of days on inotropic support - median (IQR) | 0 (0-2) | 1 (0-2) | 0 (0-2) | 0.60 |
| Number of days on vasopressor support - median (IQR) | 0 (0-3) | 0 (0-3) | 0 (0-3) | 0.66 |
| ICU Length of stay - median (IQR) | 4 (2-9) | 4 (2-10) | 4 (2-9) | 0.65 |
| > 5 days – n (%) | 986 (37.4%) | 502 (37.8%) | 484 (37.0%) | 0.69 |
| ICU mortality – n (%) | 393 (14.9%) | 217 (16.3%) | 176 (13.4%) | 0.04 |
| Hospital mortality – n (%) | 580 (22.0%) | 314 (23.6%) | 266 (20.3%) | 0.04 |

LGC, liberal glucose control; TGC, tight glucose control; ICU, intensive care unit; CRP, C-reactive protein

* Missing data on CRP peak value: n=5

**Supplementary Table S2** Demographic and clinical baseline characteristics according to surgical versus medical ICU admission

| **Characteristic** | **Total population**  **n=2639** | **SICU**  **n=1517 (57.5%)** | **MICU**  **n=1122 (42.5%)** | **P-value** |
| --- | --- | --- | --- | --- |
| Age - median (IQR) | 66 (55-74) | 66 (56-73) | 65 (54-75) | 0.19 |
| Male gender – n (%) | 1767 (67.0%) | 1076 (70.9%) | 691 (61.6%) | <0.01 |
| Diabetes mellitus – n (%) | 390 (14.8%) | 199 (13.1%) | 191 (17.0%) | <0.01 |
| Hypertension * – n (%) | 695 (28.1%) | 437 (29.8%) | 258 (25.6%) | 0.02 |
| Heart failure * – n (%) | 282 (11.4%) | 170 (11.6%) | 112 (11.1%) | 0.75 |
| Pre-existing atrial fibrillation * – n (%) | 417 (16.7%) | 208 (14.2%) | 209 (20.3%) | <0.01 |
| Body-mass index (kg/m2) * - median (IQR) | 25 (23-28) | 26 (23-29) | 24 (22-27) | <0.01 |
| APACHE II * - median (IQR) | 12 (8-18) | 9 (7-13) | 18 (14-24) | <0.01 |
| TISS-28 first 24h * - median (IQR) | 36 (29-44) | 43 (36-47) | 29 (24-33) | <0.01 |
| Blood glucose on admission (mg/dl) - median (IQR) | 137 (110-173) | 132 (107-162) | 146 (115-188) | <0.01 |

* Missing data on baseline characteristics: Hypertension: SICU n=48, MICU n=113; Heart failure: SICU n=48, MICU n=110; Pre-existing AF: SICU n=47, MICU n=92; Body-mass index: SICU n=14, MICU n=26; APACHE II: MICU n=8; TISS-28: MICU n=27

**Supplementary Table S3** Demographic and clinical baseline characteristics according to atrial fibrillation diagnosis during ICU stay

| **Characteristic** | **Total population**  **n=2639** | **No atrial fibrillation**  **n=1794 (68.0%)** | **Atrial fibrillation**  **n=845 (32.0%)** | **P-value** |
| --- | --- | --- | --- | --- |
| Age - median (IQR) | 66 (55-74) | 63 (52-72) | 71 (63-76) | <0.01 |
| Male gender – n (%) | 1767 (67.0%) | 1221 (68.1%) | 546 (64.6%) | 0.08 |
| Diabetes mellitus – n (%) | 390 (14.8%) | 244 (13.6%) | 146 (17.3%) | 0.02 |
| Hypertension * – n (%) | 695 (28.1%) | 414 (24.6%) | 281 (35.3%) | <0.01 |
| Heart failure * – n (%) | 282 (11.4%) | 151 (9.0%) | 131 (16.4%) | <0.01 |
| Pre-existing atrial fibrillation * – n (%) | 417 (16.7%) | 141 (8.3%) | 276 (34.1%) | <0.01 |
| Body-mass index (kg/m2) * - median (IQR) | 25 (23-28) | 24.7 (22-28) | 25.2 (23-29) | 0.02 |
| APACHE II * - median (IQR) | 12 (8-18) | 12 (8-17) | 14 (9-20) | <0.01 |
| TISS-28 first 24h * - median (IQR) | 36 (29-44) | 35 (28-43) | 38 (31-46) | <0.01 |
| Blood glucose on admission (mg/dl) - median (IQR) | 137 (110-173) | 136 (109-169) | 140 (112-180) | 0.02 |
| SICU – n (%) | 1517 (57.5%) | 1012 (56.4%) | 505 (59.8%) | 0.11 |
| Admission after cardiac surgery – n (%) | 956 (36.2%) | 620 (34.6%) | 336 (39.8%) | 0.01 |

APACHE II, Acute Physiology and Chronic Health Evaluation II; TISS-28, Therapeutic Intervention Scoring System-28; SICU: surgical intensive care unit

* Missing data on baseline characteristics: Hypertension: n=161; Heart failure: n=158; Pre-existing AF: n=139; Body-mass index: n=40; APACHE II: n=8; TISS-28: n=27

**Supplementary Table S4** Impact of TGC on atrial fibrillation during ICU stay – multivariable analysis

| **Baseline covariates** | **Adjusted OR (95% CI)** | **Adjusted P-value** |
| --- | --- | --- |
| Tight versus liberal glucose control | 0.92 (0.77 – 1.11) | 0.40 |
|  |  |  |
| Age (per year) | 1.03 (1.02 – 1.04) | < 0.01 |
| Sex (male versus female) | 0.92 (0.75 – 1.12) | 0.42 |
| History of diabetes mellitus | 0.95 (0.73 – 1.23) | 0.69 |
| History of hypertension | 1.24 (1.00 – 1.52) | 0.046 |
| History of heart failure | 1.24 (0.93 – 1.65) | 0.15 |
| History of atrial fibrillation | 4.16 (3.24 – 5.35) | < 0.01 |
| BMI (per kg/m^2^) | 1.02 (1.00 – 1.04) | 0.08 |
| APACHE II score (per unit change) | 1.04 (1.02 – 1.06) | < 0.01 |
| TISS-28 score first 24 hours (per unit change) | 1.03 (1.02 – 1.05) | < 0.01 |
| SICU versus MICU | 1.26 (0.92 – 1.72) | 0.15 |

The table shows the results of multivariable logistic regression analysis adjusting for the different baseline characteristics associated with the development of atrial fibrillation

BMI, body mass index; APACHE II, Acute Physiology and Chronic Health Evaluation II; TISS-28, Therapeutic Intervention Scoring System-28; SICU: surgical intensive care unit; MICU: medical intensive care unit

**Supplementary Table S5** Impact of TGC on new-onset atrial fibrillation during ICU stay – multivariable analysis

| **Baseline covariates** | **Adjusted OR (95% CI)** | **Adjusted P-value** |
| --- | --- | --- |
| Tight versus liberal glucose control | 0.92 (0.75 – 1.12) | 0.39 |
|  |  |  |
| Age (per year) | 1.02 (1.01 – 1.03) | < 0.01 |
| Sex (male versus female) | 0.96 (0.78 – 1.19) | 0.72 |
| History of diabetes mellitus | 0.88 (0.67 – 1.17) | 0.38 |
| History of hypertension | 1.12 (0.90 – 1.40) | 0.31 |
| History of heart failure | 0.95 (0.70 – 1.29) | 0.75 |
| BMI (per kg/m^2^) | 1.03 (1.00 – 1.05) | 0.02 |
| APACHE II score (per unit change) | 1.04 (1.02 – 1.05) | < 0.01 |
| TISS-28 score first 24 hours (per unit change) | 1.03 (1.02 – 1.05) | < 0.01 |
| SICU versus MICU | 1.36 (0.98 – 1.91) | 0.07 |

The table shows the results of multivariable logistic regression analysis adjusting for the different baseline characteristics associated with the development of atrial fibrillation

BMI, body mass index; APACHE II, Acute Physiology and Chronic Health Evaluation II; TISS-28, Therapeutic Intervention Scoring System-28; SICU: surgical intensive care unit; MICU: medical intensive care unit

**Supplementary Figure S1** Impact of TGC on new-onset atrial fibrillation during ICU stay in prespecified subgroups


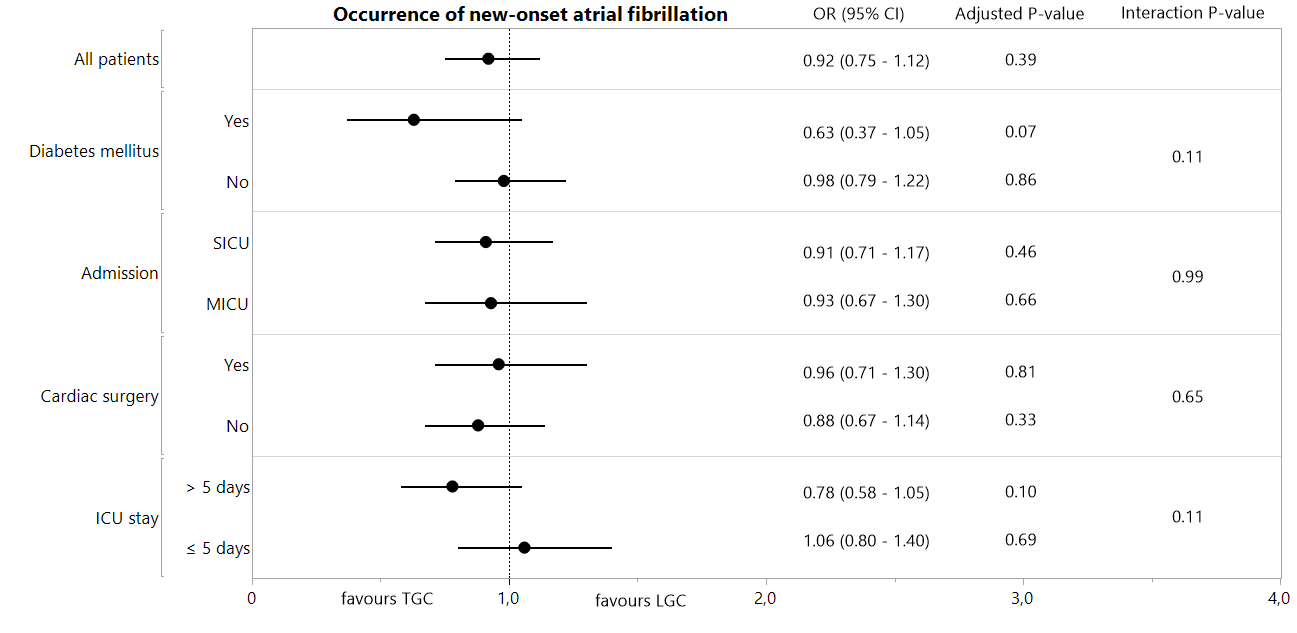


The panel shows a forest plot of the impact of tight glucose control versus liberal glucose control in subgroups with respect to the development of new-onset atrial fibrillation during ICU stay

Odds ratios and 95% confidence intervals, P-values and interaction P-values are all adjusted analyses

SICU: surgical intensive care unit; MICU: medical intensive care unit; ICU: intensive care unit

**Supplementary Table S6** Association of atrial fibrillation during ICU stay with ICU morbidity and short-term outcomes

| **Outcome parameter** | **Total population**  **n=2639** | **No atrial fibrillation**  **n=1794 (68.0%)** | **Atrial fibrillation**  **n=845 (32.0%)** | **P-value** |
| --- | --- | --- | --- | --- |
| Duration of ventilatory support during ICU stay - median (IQR) | 2 (1-9) | 2 (1-6) | 5 (2-12) | <0.01 |
| Renal impairment during ICU stay |  |  |  |  |
| Peak creatinine value (mg/dl) - median (IQR) | 1.20 (0.94-1.97) | 1.13 (0.90-1.65) | 1.47 (1.07-2.92) | <0.01 |
| Renal replacement therapy – n (%) | 327 (12.4%) | 136 (7.6%) | 191 (22.6%) | <0.01 |
| CRP peak value* (mg/dl) - median (IQR) | 186 (106-266) | 168 (88-248) | 219 (148-287) | <0.01 |
| ICU bacteremia – n (%) | 179 (6.8%) | 91 (5.0%) | 88 (10.4%) | <0.01 |
| Number of days on inotropic support - median (IQR) | 0 (0-2) | 0 (0-2) | 2 (0-4) | <0.01 |
| Number of days on vasopressor support - median (IQR) | 0 (0-3) | 0 (0-2) | 2 (0-5) | <0.01 |
| ICU Length of stay - median (IQR) | 4 (2-9) | 3 (2-7) | 6 (3-15) | <0.01 |
| > 5 days – n (%) | 986 (37.4%) | 559 (31.2%) | 427 (50.5%) | <0.01 |
| ICU mortality – n (%) | 393 (14.9%) | 194 (10.8%) | 199 (23.6%) | <0.01 |
| Hospital mortality – n (%) | 580 (22.0%) | 304 (17.0%) | 276 (32.7%) | <0.01 |

ICU: intensive care unit; CRP, C-reactive protein

* Missing data on CRP peak value: n=5
